# Lack of Overt Retinal Degeneration in a K42E *Dhdds* Knock-In Mouse Model of RP59

**DOI:** 10.3390/cells9040896

**Published:** 2020-04-07

**Authors:** Sriganesh Ramachandra Rao, Steven J. Fliesler, Pravallika Kotla, Mai N. Nguyen, Steven J. Pittler

**Affiliations:** 1 Research Service, VA Western NY Healthcare System, Buffalo, NY 14215, USA; sramacha@buffalo.edu (S.R.R.); fliesler@buffalo.edu (S.J.F.); 2 Departments of Ophthalmology and Biochemistry and Neuroscience Graduate Program, The State University of New York- University at Buffalo, Buffalo, NY 14209, USA; 3 Department of Optometry and Vision Science, Vision Science Research Center, University of Alabama at Birmingham, School of Optometry, Birmingham, AL 35294, USA; pkotla@uab.edu (P.K.); mnnguyen@uab.edu (M.N.N.)

**Keywords:** retinitis pigmentosa, knock-in mouse model, congenital disorder of glycosylation, retina

## Abstract

Dehydrodolichyl diphosphate synthase (DHDDS) is required for protein *N*-glycosylation in eukaryotic cells. A K42E point mutation in the DHDDS gene causes an autosomal recessive form of retinitis pigmentosa (RP59), which has been classified as a congenital disease of glycosylation (CDG). We generated K42E *Dhdds* knock-in mice as a potential model for RP59. Mice heterozygous for the *Dhdds* K42E mutation were generated using CRISPR/Cas9 technology and crossed to generate *Dhdds*^K42E/K42E^ homozygous mice. Spectral domain-optical coherence tomography (SD-OCT) was performed to assess retinal structure, relative to age-matched wild type (WT) controls. Immunohistochemistry against glial fibrillary acidic protein (GFAP) and opsin (1D4 epitope) was performed on retinal frozen sections to monitor gliosis and opsin localization, respectively, while lectin cytochemistry, plus and minus PNGase-F treatment, was performed to assess protein glycosylation status. Retinas of *Dhdds*^K42E/K42E^ mice exhibited grossly normal histological organization from 1 to 12 months of age. Anti-GFAP immunoreactivity was markedly increased in *Dhdds*^K42E/K42E^ mice, relative to controls. However, opsin immunolocalization, ConA labeling and PNGase-F sensitivity were comparable in mutant and control retinas. Hence, retinas of *Dhdds*^K42E/K42E^ mice exhibited no overt signs of degeneration, yet were markedly gliotic, but without evidence of compromised protein *N*-glycosylation. These results challenge the notion of RP59 as a DHDDS loss-of-function CDG and highlight the need to investigate unexplored RP59 disease mechanisms.

## 1. Introduction

Retinitis pigmentosa (RP) represents a group of hereditary retinal degenerative disorders of diverse genetic origins that have as their common trait the progressive, irreversible dysfunction, degeneration, and demise of retinal photoreceptor cells, with rods initially undergoing these pathological changes followed eventually by cones [1,2]. Relatively recently, a K42E point mutation in the dehydrodolichyl diphosphate synthase (DHDDS) gene was shown to cause a rare, recessive form of RP (RP59; OMIM #613861) [3,4,5]. DHDDS catalyzes *cis*-prenyl chain elongation in the synthesis of dolichyl diphosphate (Dol-PP), which is required for protein *N*-glycosylation [6,7]. DHDDS catalyzes the condensation of multiple units of isopentenyl pyrophosphate (IPP, also called isopentenyl diphosphate) to farnesyl pyrophosphate (FPP, also called farnesyl diphosphate) to produce Dol-PP [8,9]. This is used as the “lipid carrier” onto which oligosaccharide chains are built that are ultimately transferred to specific asparagine (*N*) residues on nascent polypeptide chains in the lumen of the endoplasmic reticulum (ER) to form *N*-linked glycoproteins [10]. The monophosphate (Dol-P) is used as a sugar carrier, transferring sugars from their corresponding sugar-nucleotide adducts (e.g., UDP-glucose, GDP-mannose, etc.) to the growing Dol-PP-linked oligosaccharide chains in the ER. Mutations in rhodopsin that block its glycosylation have been shown to cause retinal degeneration in vertebrate animals [11,12]. In addition, pharmacological inhibition of protein *N*-glycosylation with tunicamycin has been shown to disrupt retinal photoreceptor outer segment (OS) disc membrane morphogenesis in vitro [13], as well as to cause retinal degeneration with progressive shortening and loss of photoreceptor OSs in vivo [14].

In the present study, we created a DHDDS K42E homozygous knock-in mouse model (hereafter called *Dhdds*^K42E/K42E^) of RP59—since K42E is the most prevalent point mutation in the RP59 patient population [3,4,5]—to study its underlying pathological mechanism, with the working hypothesis that defective protein *N*-glycosylation underlies the retinal dysfunction and degeneration observed in human RP59. Herein, we present a description of the generation and initial characterization of the phenotypic features of the *Dhdds*^K42E/K42E^ mouse model. Surprisingly, although we expected to observe an early onset, progressive, and potentially severe retinal degeneration, this was not the case. The retina appeared histologically intact and normal according to spectral domain optical coherence tomography (SD-OCT) analysis for up to at least one year of age. However, there was evidence of gliotic reactivity (glial fibrillary acidic protein (GFAP) immunostaining), despite the lack of obvious neuronal degeneration or cell death/loss. Also, despite the homozygous mutation in *Dhdds*, we found no evidence of compromised protein *N*-glycosylation in mutant mouse retinas.

## 2. Materials and Methods

### 2.1. Animals

Heterozygous (K42E/+) *Dhdds* knock-in (KI) mice were generated on a C57Bl/6J background by Applied StemCell (Milipitas, CA, USA). Briefly, CRISPR guide RNA (5′-TCGCTATGCCAAGAAGTGTC-3′ with PAM site AGG) was generated using in vitro transcription and was used to create a double strand break in the murine *Dhdds* locus to promote introduction of a single-stranded oligodeoxynucleotide (SSO) carrying the K42E mutation and a second silent DNA polymorphism to eliminate the PAM recognition site required for cleavage by CAS9 (5′-ATTATCTGTTCTCTTCTACAGGCTGGCCCAGTACCCAAACATATCGCGTTCATAATGGACGGCAACCGTCGCTATGCCAAGGAGTGTCAAGTGGAGCGCCAGGAGGGCCACACACAGGGCTTCAATAAGCTTGCTGAGGTGGGTGCGGGTGACAGAGCCTAGA-3′). Mouse zygotes were injected with 100, 100, and 250 ng/μL of Cas9 enzyme, guide RNA, and SSO, respectively, which were then transferred into pseudo pregnant CD-1 females. Three potential founder (F0) pups were identified out of 13 mice tested, and an F0 founder was verified by DNA sequence analysis. Sequence-validated heterozygous (*Dhdds*^K42E/+^) mice were crossed to generate homozygous (*Dhdds*^K42E/K42E^) mice, as confirmed by PCR and DNA sequencing (see below). C57Bl/6J wild type (WT) mice, age- and sex-matched, were used as controls. All procedures conformed to the ARVO Statement for the Use of Animals in Ophthalmic and Vision Research, and were approved by the Institutional Animal Care and Use Committee (IACUC) of the University of Alabama at Birmingham. All animals were maintained on a standard 12/12 h light/dark cycle (20–40 lux ambient room illumination), fed standard rodent chow, provided water ad libitum, and housed in plastic cages with standard rodent bedding.

### 2.2. PCR Genotyping and DNA Analysis

PCR primers were designed that spanned the targeted region (forward primer, 5′-TCTAGGCTCTGTCACCCGCA-3′ and reverse primer 5′-TCTAGGCTCTGTCACCCGCA-3′) amplifying a 292 bp segment of DNA in both WT and *Dhdds*^K42E/K42E^ mice. For initial verification of the knock-in, PCR products were sequenced in the UAB Heflin Center for Genomic Sciences. The presence of the knock-in sequence was confirmed in subsequent generations by restriction enzyme digestion with StyI, which cleaves the knock-in allele only (data not shown). Knock-in alleles were independently verified by Transnetyx, Inc. (Cordova, TN, USA) using proprietary technology. While the analysis was set up to recognize and differentiate the knock-in mutation and the PAM site polymorphism, only the knock-in mutation was maintained in all subsequent breeding.

### 2.3. Spectral Domain Optical Coherence Tomography (SD-OCT)

*In vivo* retinal imaging was performed as previously described in detail by DeRamus et al. [15], using a Bioptigen Model 840 Envisu Class-R high-resolution SD-OCT instrument (Bioptigen/Leica, Inc.; Durham, NC, USA). Data were collected from *Dhdds*^K42E/K42E^ and WT mice at postnatal day (PN) 1 (KI, n = 5; WT, n = 9), 2 (KI, n = 4; WT, n = 8), 3 (KI, n = 5; WT n = 3), 8 (KI, n = 4; WT n = 5), and 12 months (mos) (KI, n = 3; WT n = 3) to assess retinal structure. Layer thicknesses were determined manually using Bioptigen InVivoVue^®^ and Bioptigen Diver^®^ V. 3.4.4 software and the data were analyzed and graphed using Microsoft Excel software. 

### 2.4. Immunohistochemistry (IHC)

Procedures utilized for fixation, O.C.T. embedment, and sectioning of mouse eyes were as described in detail previously by Ramachandra Rao et al. [16]. In brief, eyes were immersion fixed overnight in phosphate-buffered saline (PBS) containing freshly prepared paraformaldehyde (4% v/v), appropriately cryopreserved, embedded in O.C.T., and cryosectioning was performed on a Leica Model CM3050 S Cryostat (Leica Biosystems, Wetzlar, Germany). Retinal sections were first “blocked” with 0.1% BSA, 0.5% serum (species corresponding to secondary antibody host) in Tris-buffered saline containing 0.1% Tween-20 (TBST), then incubated for 1 h at room temperature with a rabbit polyclonal antibody against glial fibrillary acidic protein (GFAP;, DAKO/Agilent, Santa Clara, CA, USA; 1:500 dilution in TBST) and a mouse monoclonal antibody against the C-terminal epitope of opsin (1D4; Novus Biologicals, Littleton, CO, USA; 1:500 dilution in TBST), followed by incubation with fluor-conjugated secondary antibodies (AlexaFluor^®^-488 conjugated anti-mouse IgG, AlexaFluor^®^-568 conjugated anti-rabbit IgG; Thermo Fisher Scientific, Waltham, MA, USA; 1:500 dilution in TBST). Sections were then counterstained with DAPI and cover slipped with anti-fade mounting medium (Vectashield^®^; Vector Laboratories, Burlingame, CA, USA) and viewed with a Leica TCS SPEII DMI4000 scanning laser confocal microscope (Leica Biosystems). Images were captured using a 40X oil immersion (RI 1.518) objective under normal laser intensity (10% of laser power source), arbitrary gain (850 V) and offset (–0.5) values, to optimize signal-to-noise ratio. Digital images were captured and stored as TIFF files on a PC computer.

### 2.5. Lectin Cytochemistry

Paraformaldehyde-fixed eyes (as described above) were processed for paraffin embedment. Paraffin sections of mouse eyes were then incubated (45 min at room temperature) with biotinylated Concanavalin-A (ConA, B-1005; Vector Laboratories; 1:200 dilution in PBS), followed by incubation with AlexaFluor^®^-488 conjugated streptavidin (Thermo Fisher Scientific; 1:500 dilution in PBS) and AlexaFluor^®^-647-conjugated peanut agglutinin (PNA, L32460; Thermo Fisher Scientific; 1:250 dilution in PBS), with or without pre-treatment (37 °C, overnight) with peptide:N-glycosidase F (PNGase-F, 200 U, P0704S; New England Biolabs, Inc., Ipswich, MA, USA). Sections were DAPI-stained and mounted using Vectashield mounting media, and digital images obtained using scanning laser confocal microscopy as described above [16].

## 3. Results

### 3.1. Generation and Validation of K42E DHDDS Knock-In Mutation

**Figure 1:**
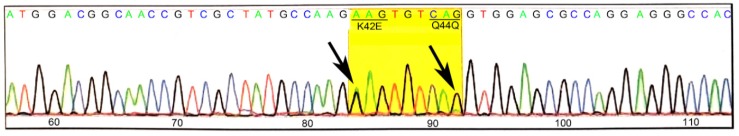
DNA sequence analysis of a tail DNA from a K42E/+ founder mouse. Tail DNA was amplified with primers that cover a 292 bp segment spanning the target region. The sequence analysis confirmed the presence (arrows) of the K42E (A-to-G) mutation and the Q44Q (G-to-A) polymorphism that was included to eliminate the CRISPR-related PAM site.

K42E knock-in mice were generated commercially using CRISPR-Cas9 technology. The K42E knock-in mutations in both heterozygous and homozygous mice were confirmed by DNA sequence for one of the heterozygous F0 founder mice, which is shown in Figure 1. Both the A-to-G and G-to-A transitions that lead to the K42E mutation and the Q44Q silent polymorphism, respectively, are heterozygous (arrows). Intra-litter mating was done to establish at least fourth generation homozygous mice that were used for all subsequent analyses. Heterozygous mice were initially characterized by SD-OCT and histology and found not to differ from WT (not shown).

### 3.2. SD-OCT Analysis Reveals No Evidence for Retinal Degeneration in Dhdds^K42E/K42E^ Mice

**Figure 2:**
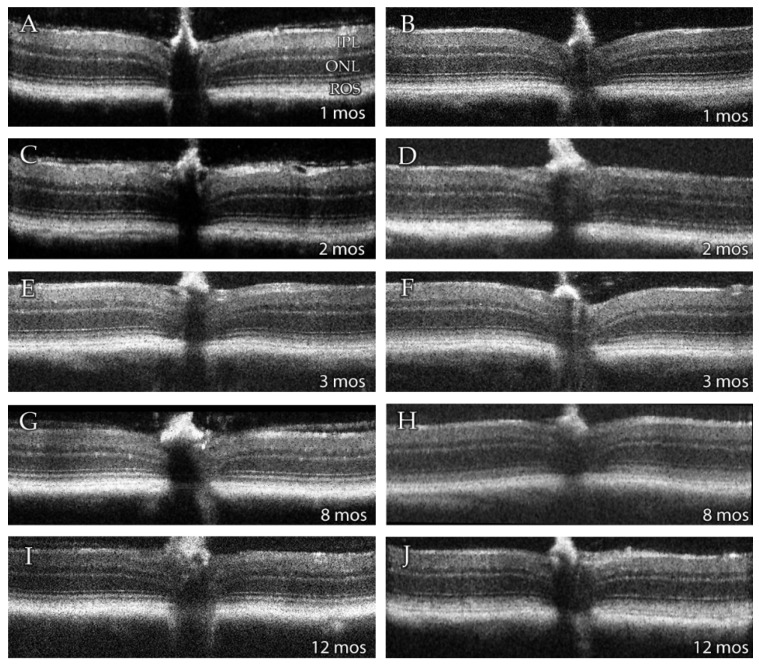
Representative averaged SD-OCT images of retinas from (left panels: **A**,**C**,**E**,**G**,**I**) 1-, 2-, 3-, 8- and 12-months (mos) old wild type (WT), and (right panels: **B**,**D**,**F**,**H**,**J**) 1-, 2-, 3-, 8-, and 12-months old *Dhdds*^K42E/K42E^ mice. Abbreviations: IPL, inner plexiform layer; ONL, outer nuclear layer; ROS, rod outer segment layer. No changes were observed at any age in retinas of *Dhdds*^K42E/K42E^ mice compared to WT mice.

SD-OCT provides a non-invasive means of assessing retinal morphology *in vivo*. Qualitative SD-OCT images obtained from wild type (WT) and *Dhdds*^K42E/K42E^ mice are presented in Figure 2. From these images, it is clear that the gross morphology of the retina in the homozygous knock-in animals, from PN 1 to 12 months of age, are comparable to that observed in fully mature, age-matched WT control mice. All retinal histological layers were intact and of normal appearance. Hence, there was no evidence of retinal degeneration, even up to one year of age.

**Figure 3:**
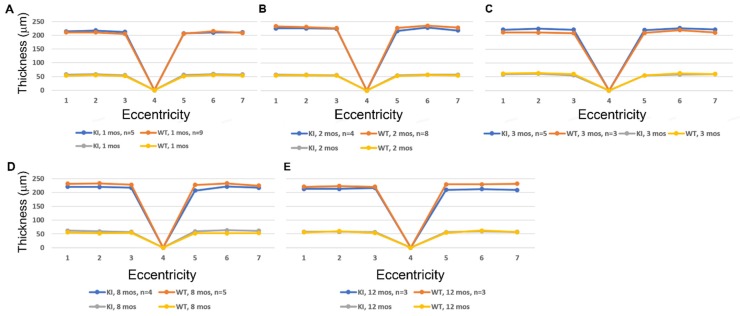
Analysis of the ONL thickness (yellow and gray lines) and total retinal thickness (blue and orange lines) in WT and *Dhdds*^K42E/K42E^ mice ranging in age from PN 1 to 12 months. (**A**) 1 month, (**B**) 2 months, (**C**) 3 months, (**D**) 8 months, (**E**) 12 months. Outer nuclear layer (ONL) thickness and total retina thickness measurements (in microns), as a function of genotype and distance from the optic nerve head (ONH) along the vertical meridian in both the inferior and superior hemispheres. Genotypes: WT and *Dhdds*^K42E/K42E^ mice. No significant differences were observed between the groups.

We used SD-OCT to perform quantitative analysis of retinal morphology to compare ocular tissue layer thicknesses in WT and knock-in mice. Figure 3 compares data obtained at PN 1, 2, 3, 8, and 12 mos for *Dhdds*^K42E/K42E^ mice, compared to age-matched WT control littermates. The data are shown both with respect to outer nuclear layer (ONL) thickness (yellow and gray lines) as well as total neural retina thickness (blue and orange lines) as a function of distance from the optic nerve head (ONH, point 4 in each graph) along the vertical meridian, for both the inferior and superior hemispheres. No differences in these quantitative metrics of retinal morphology were observed with respect to genotype, consistent with the representative OCT images shown in Figure 2.

### 3.3. Gliotic Reactivity, Despite Lack of Overt Neural Retina Degeneration, in Dhdds^K42E/K42E^ Mice

**Figure 4:**
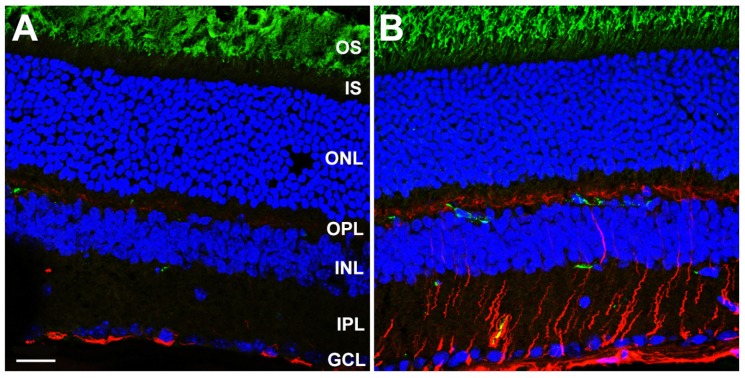
Laser confocal microscopy images of (**A**) WT control and (**B**) *Dhdds*^K42E/K42E^ mouse retina frozen sections at PN 2 months of age, stained with antibodies to GFAP (pseudocolor: red) and rod opsin (pseudocolor: green), and counterstained with DAPI (blue). Scale bar (both panels) is 20 μm. Abbreviations: OS, outer segment layer; IS, inner segment layer; ONL, outer nuclear layer; OPL, outer plexiform layer; INL, inner nuclear layer; IPL, inner plexiform layer; GCL, ganglion cell layer. Scale bar (both panels) is 20 μm.

We performed immunohistochemical analysis on frozen sections of fixed, O.C.T.-embedded WT and *Dhdds*^K42E/K42E^ mouse eyes at PN 2 months of age, probing with antibodies against GFAP (polyclonal) and a C-terminal epitope of rod opsin (1D4 monoclonal). As shown in Figure 4, whereas WT control retinas only exhibited GFAP immunoreactivity (pseudocolored red) along the vitreoretinal interface, corresponding to astrocytes and Müller glia “end feet”, retinas from *Dhdds*^K42E/K42E^ mice exhibited extensive, robust anti-GFAP labeling in a radial pattern. This extended throughout the inner retinal layers to the outer plexiform layer (OPL), in addition to intense labeling along the vitreoretinal interface. The latter results are indicative of massive gliotic activation, which is remarkable considering the lack of overt retinal degeneration or loss of retinal neurons (per the SD-OCT data; see Figure 2 and Figure 3). Gliosis in *Dhdds*^K42E/K42E^ mouse retinas was also detected at PN one month and persisted even at PN six months of age (data not shown). Anti-opsin immunolabeling (pseudocolored green) was comparable in both WT and *Dhdds*^K42E/K42E^ retinas. Notably, the label was confined to the OS layer; there was no mislocalization of opsin to the plasma membrane of the cell in the IS or ONL layer—unlike what is often observed in degenerating photoreceptor cells in various animal models—suggesting normal trafficking of opsin to the outer segment, and consistent with a lack of overt photoreceptor degeneration. It is worth noting that the green labeling in a few cells in the inner retina in Figure 4 is due to mouse-on-mouse binding of the monoclonal antibody to endogenous IgG in blood vessels. It does not represent true anti-opsin immunolabeling.

### 3.4. Lack of Defective Protein Glycosylation in Dhdds^K42E/K42E^ Mouse Retinas

**Figure 5:**
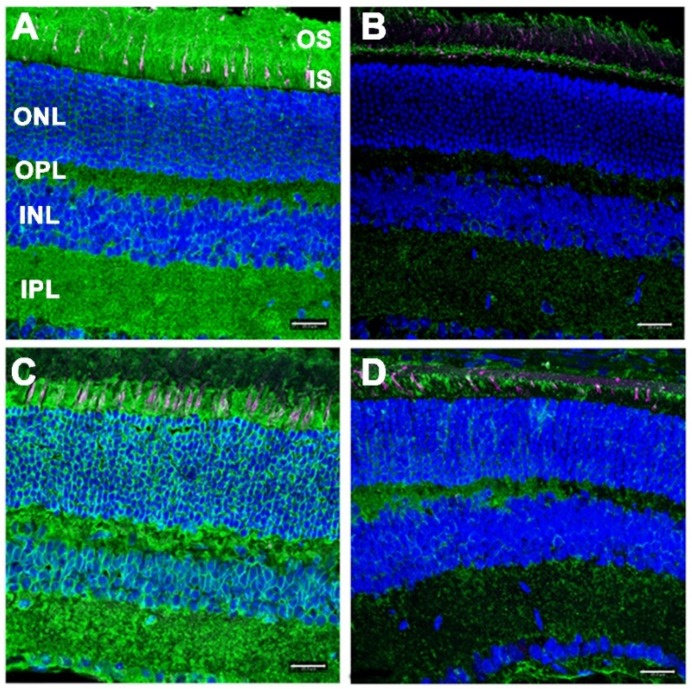
ConA lectin cytochemical analysis of retinas from (**A**,**B**) WT control and (**C**,**D**) *Dhdds*^K42E/K42E^ mice at PN 6 months of age, with (**B**,**D**) or without (**A**,**C**) pretreatment with PNGase-F. ConA binding (green); PNA binding (magenta); DAPI counterstain (blue). Abbreviations are the same as in the Figure 4 legend. Scale bar (all panels): 20 μm.

The *N*-linked oligo-saccharides of glycoproteins contain alpha-linked mannose residues as constituents, which are cognate ligands for the lectin concanavalin A (Con A) [17]. Hence, ConA lectin cytochemistry offers a reliable means of detecting the presence (or absence) of N-linked oligo-saccharides in tissue sections of *Dhdds*^K42E/K42E^ mice, and a way to directly test the current hypothesis that RP59 is driven by lack of glycosylation. This is because the synthesis of oligosaccharide chains in cells and tissues obligatorily depends upon the presence of Dol-PP and Dol-P (which requires upstream DHDDS activity). Furthermore, N-linked oligosaccharide chains are selectively susceptible to hydrolysis by peptide:N-glycosidase F (PNGase-F) [18]; hence, tissue sections treated with PNGase-F should exhibit a marked loss of Con A binding (serving as a true negative control), thereby mimicking the scenario where upstream DHDDS activity may be lacking. We performed ConA lectin cytochemical analysis on retinal sections from WT control and *Dhdds*^K42E/K42E^ mice at PN six months of age, with and without pre-treatment with PNGase-F. The results are shown in Figure 5.

Normally, *N*-linked glycoproteins are present throughout the retina, being notably enriched in photoreceptor cells and the synaptic endings of neurons (IPL, OPL). Hence, the inner and outer segment layers (IS and OS, respectively), including the glycoconjugate-rich interphotoreceptor matrix (IPM), as well as the inner and outer plexiform layers (IPL and OPL, respectively) were robustly labeled with fluor-tagged ConA in untreated WT retinal sections (Figure 5 A). As expected, treatment of WT retinal tissue sections with PNGase-F dramatically reduced the level of ConA binding throughout the retina (Figure 5B). Notably, retinal sections from *Dhdds*^K42E/K42E^ mice also exhibited robust, pan-retinal ConA binding (Figure 5C), comparable to that of WT controls. Upon treatment with PNGase-F, most of the ConA staining was lost (Figure 5D). These results obviate any significant DHDDS loss-of-function in *Dhdds*^K42E/K42E^ mice.

Peanut agglutinin (PNA) binds to the disaccharide Gal-β(1-3)-GalNAc in glycoproteins and glycolipids [17]. Oligosaccharides containing this disaccharide are highly enriched in the extracellular matrix surrounding cone photoreceptor outer segments (the “cone matrix sheath”) [19,20]. Thus, PNA binding can be used to selectively label cone photoreceptors in retinal tissue sections, since rod photoreceptors and their associated “rod matrix sheath” lack such glycan chains [21]. Furthermore, oligosaccharides containing Gal-β(1-3)-GalNAc are generally *O*-linked (e.g., through Ser or Thr residues), rather than *N*-linked, and their synthesis is not dolichol-dependent in mammalian cells [22]. Furthermore, the PNA-binding disaccharide epitope is not susceptible to PNGase-F hydrolysis [17]. Hence, we expected to observe no appreciable differences in the binding of PNA to retinal tissue sections from *Dhdds*^K42E/K42E^ retinas vs. WT controls, nor effects of PNGase-F treatment on PNA binding, either with regard to labeling intensity or distribution. These expectations were realized, as illustrated in Figure 5 (magenta staining, all four panels). Both *Dhdds*^K42E/K42E^ and control retinas exhibited comparable distribution of PNA-positive cone matrix sheaths, suggesting persistence of viable cone photoreceptors in the K42E mutants.

## 4. Discussion

Here, we have presented the generation and initial characterization of a novel mouse model of RP59, where we have achieved global homozygous knock-in of the *K42E Dhdds* mutation specifically associated with RP59 [3,4,5]. Based upon the clinical presentation of RP59 in human patients [3,4,5], as well as the demonstrable importance of dolichol-dependent protein glycosylation in maintaining the normal structure and function of the vertebrate retina [11,12,13,14], we expected to observe retinal degeneration and retinal thinning in *Dhdds*^K42E/K42E^ mice, particularly in mice homozygous for the K42E mutation. This expectation was also predicated on a preliminary report [23], using a similar K42E mouse knock-in model, that claimed nearly 50% loss of OS length and reduction in ONL thickness by about two-thirds at PN 3 months of age, compared to WT mouse retinas. However, we observed no evidence of retinal degeneration in *Dhdds*^K42E/K42E^ mice up to one year of age. Furthermore, despite the confirmed mutation of *Dhdds*, we found no evidence for defective protein *N*-glycosylation in the retinas of these mice. The retinas were labeled robustly with fluor-tagged ConA lectin, irrespective of genotype. These findings are in good agreement with observations made by Sabry et al. [24] who found normal mannose incorporation into *N*-linked oligosaccharides using either siRNA silencing of *DHDDS* in a HepG2 cell line or in RP59 (severe mutation) patient fibroblasts. The ConA binding observed in our study is further consistent with observations made by Wen et al., who observed that rather than any loss in dolichols, there was an alteration in dolichol chain lengths (increased D17:D18 ratio) in RP59 patients compared to normal human subjects, but without obvious hypoglycosylation of serum transferrin [25]. These findings collectively suggest hypoglycosylation-independent retinal degeneration in RP59, the mechanism of which still remains to be elucidated.

Understanding the pathophysiological and biochemical mechanisms underlying RP59 remains limited due to the lack of a validated vertebrate animal model that faithfully mimics the key hallmarks of the disease. Heretofore, only a zebrafish model of RP59 has been documented, using global knock-down of *DHDDS* expression by injection of morpholino oligonucleotides at the one-cell embryo stage [26]. In that case, the fish exhibited defective photoresponses and their cone outer segments (as assessed indirectly by PNA staining) were dramatically shortened, if not nearly absent. It should be noted that zebrafish have a highly cone-rich retina, unlike humans or mice (which have highly rod-dominant retinas). Also, the reduction and loss of PNA binding in the zebrafish knock-down retinas most likely reflects degeneration and death of cone photoreceptors, with concomitant degeneration and loss of their outer segments, due to their requirement for dolichol. Unlike the zebrafish *Dhdds* knock-down model, the murine RP59 model generated in the present study exhibits robust PNA staining in the outer retina, suggesting persistence of viable cone photoreceptors. In a parallel study (Ramachandra Rao et al., unpublished), we have observed *Dhdds* transcript distribution in all retinal nuclear layers by in situ hybridization, consistent with the fact that all cells require dolichol derivatives to support protein *N*-glycosylation. Taken together, these findings suggest that the K42E *Dhdds* mutation does not affect cone photoreceptor viability. Recently (Ramachandra Rao et al., manuscript submitted for publication), we also generated a conditional *Dhdds* knockout mouse model, with targeted ablation of *Dhdds* in retinal rod photoreceptors, using a Cre-lox approach; however, unlike the K42E knock-in model, the rod-specific *Dhdds* knockout model exhibits profound, rapid retinal degeneration, with almost complete loss of photoreceptors by PN 6 weeks. Yet, there was no evidence of compromised protein *N*-glycosylation prior to the onset of photoreceptor degeneration. In addition, as reported in a companion article in this Special Issue of *Cells* [27], targeted ablation of *Dhdds* in retinal pigment epithelium, (RPE) cells in mice also results in a progressive, but somewhat slower, retinal degeneration.

As pointed out by Zelinger et al. [4], the phenotype of RP59 only involves the retina; there is no observable dysfunction or pathology in other tissues and organs in RP59 patients. Hence, those authors speculated that the K42E mutation, “alters, rather than abolishes, enzymatic function, perhaps either by reducing the level of DHDDS protein or by preventing requisite interactions between DHDDS and a photoreceptor-specific protein” [4]. They also suggested, alternatively, that mutation of DHDDS might result in, “a toxic accumulation of isoprenoid compounds,” such as occurs in various forms of neuronal ceroid lipofuscinosis (e.g., Batten disease). While such speculations may turn out to be true, there is no direct empirical evidence extant to support this hypothesis. It is also entirely possible, however, that mutations (whether K42E or others) in DHDDS may affect its interactions with its enzymatic partner, Nogo-B receptor (NgBR, encoded by the *Nus1* gene) [8,9], with concomitant alterations in dolichol synthesis and protein *N*-glycosylation [28,29]. At present, nothing is known about the expression of Nogo-B receptor or its interactions with DHDDS, specifically in the retina. Our *Dhdds*^K42E/K42E^ mouse line and retinal cell type-specific conditional DHDDS knockout mice offer potentially valuable model systems in which to pursue further investigations along these lines. In addition, we are currently pursuing studies employing dual, targeted ablation of DHDDS and NgBR in the retina. (See also the article by DeRamus et al., in this Special Issue of *Cells*, regarding an RPE-specific DHDDS knockout mouse model [27].)

Our findings bring into question the current concept that RP59 is a member of a large and diverse class of diseases known as “congenital disorders of glycosylation” (CDGs) [30,31]. While, in principle, it would be reasonable to consider RP59 as a CDG, due to the associated mutation(s) in DHDDS, there is no direct evidence to demonstrate a glycosylation defect in the human retinal disease or in any animal model of RP59 generated to date. The mechanism underlying the DHDDS-dependent retinal degeneration in human arRP patients remains to be elucidated, but is more complex than simply loss-of-function of DHDDS.
